# Rethinking Lupus Nephritis Classification on a Molecular Level

**DOI:** 10.3390/jcm8101524

**Published:** 2019-09-23

**Authors:** Salem Almaani, Stephenie D. Prokopec, Jianying Zhang, Lianbo Yu, Carmen Avila-Casado, Joan Wither, James W. Scholey, Valeria Alberton, Ana Malvar, Samir V. Parikh, Paul C. Boutros, Brad H. Rovin, Heather N. Reich

**Affiliations:** 1 Division of Nephrology, The Ohio State University, Columbus, OH 43210, USASamir.Parikh@osumc.edu (S.V.P.); rovin.1@osu.edu (B.H.R.); 2 Ontario Institute for Cancer Research, Toronto, ON M5G 0A3, Canada; Stephenie.Prokopec@oicr.on.ca (S.D.P.); PBoutros@mednet.ucla.edu (P.C.B.); 3 Division of Biostatistics, Department of Information Sciences, City of Hope National Medical Center, Duarte, CA 91010, USA; jianzhang@coh.org; 4 Department of Biomedical Informatics, College of Medicine, The Ohio State University, Columbus, OH 43210, USA; Lianbo.Yu@osumc.edu; 5 Department of Pathology, Toronto General Hospital, University Health Network, Toronto, ON M5G 2C4, Canada; carmen.avila-casado@uhn.ca; 6 Division of Rheumatology, University Hospital Network, Toronto, ON M5G 2C4, Canada; Joan.Wither@uhnresearch.ca; 7 Division of Nephrology, Department of Medicine, Toronto General Hospital, University Health Network, Toronto, ON M5G 2C4, Canada; james.scholey@utoronto.ca; 8 Hospital Fernandez, Buenos Aires M5S 1A8, Argentina; vgalberton@yahoo.com.ar (V.A.); avmperrin@yahoo.com.ar (A.M.); 9 Department of Pharmacology & Toxicology, University of Toronto, Toronto, ON M5G 1L7, Canada; 10 Department of Medical Biophysics, University of Toronto, Toronto, ON M5G 1L7, Canada; 11 Department of Human Genetics, University of California, Los Angeles, CA 90095, USA; 12 Department of Urology, University of California, Los Angeles, CA 90024, USA; 13 Institute for Precision Health, University of California, Los Angeles, CA 90095, USA; 14 Jonsson Comprehensive Cancer Center, University of California, Los Angeles, CA 90024, USA

**Keywords:** lupus nephritis, ISN/RPS classification, mRNA

## Abstract

The International Society of Nephrology/Renal Pathology Society (ISN/RPS) lupus nephritis (LN) classification is under reconsideration, given challenges with inter-rater reliability and resultant inconsistent relationship with treatment response. Integration of molecular classifiers into histologic evaluation can improve diagnostic precision and identify therapeutic targets. This study described the relationship between histological and molecular phenotypes and clinical responses in LN. Renal compartmental mRNA abundance was measured in 54 biopsy specimens from LN patients and correlated to ISN/RPS classification and individual histologic lesions. A subset of transcripts was also evaluated in sequential biopsies of a separate longitudinal cohort of 36 patients with paired samples obtained at the time of flare and at follow up. Unsupervised clustering based on mRNA abundance did not demonstrate a relationship with the (ISN/RPS) classification, nor did univariate statistical analysis. Exploratory analyses suggested a correlation with individual histologic lesions. Glomerular FN1 (fibronectin), SPP1 (secreted phosphoprotein 1), and LGALS3 (galectin 3) abundance correlated with disease activity and changed following treatment. Exploratory analyses suggested relationships between specific transcripts and individual histologic lesions, with the important representation of interferon-regulated genes. Our findings suggested that the current LN classification could be refined by the inclusion of molecular descriptors. Combining molecular and pathologic kidney biopsy phenotypes may hold promise to better classify disease and identify actionable treatment targets and merits further exploration in larger cohorts.

## 1. Introduction

Kidney involvement in systemic lupus erythematosus (SLE) is a major determinant of morbidity and mortality [1]; controlling renal inflammation and preventing chronic damage attenuate mortality [2]. Therapeutic decisions are generally based on the histologic pattern of injury in kidney tissue obtained via biopsy. Currently available non-invasive clinical tests cannot distinguish histologic subtypes of lupus nephritis (LN), nor reliably differentiate active disease from chronic damage. Therefore, despite procedure-associated risks, biopsy remains the gold standard for diagnosis and treatment. Given the disconnect between non-invasive clinical tests and kidney histology, repeat biopsies are sometimes needed to evaluate kidney parenchymal changes caused by therapy, distinguish residual inflammatory activity from irreversible chronic injury, and characterize disease relapse after renal remission.

Several descriptive schemas of renal histology have been proposed to provide clinically relevant insights into LN. The most widely used are the 2004 International Society of Nephrology and the Renal Pathology Society (ISN/RPS) classification [3], and the NIH activity and chronicity indices [4]. The ISN/RPS classification is largely based on glomerular lesions, has limited short-term prognostic value [3,5], modest inter-observer reproducibility [6], and does not incorporate histologic variables known to have significant long-term prognostic implications, such as the presence of tubulointerstitial (TI) and vascular lesions [7]. The National Institute of Health (NIH) activity and chronicity indices use composite scores to quantitate and describe the collective degree of disease activity and chronic damage. These indices also have modest reproducibility and prognostic value [8,9]. Reconsideration of these classification schemas is underway, given these concerns [10]. No classification scheme incorporates information on the molecular processes responsible for the specific pattern of injury despite the emerging application of molecular profiling in SLE, and the identification of candidate molecular signatures of disease activity [11]. Molecular data are increasingly being incorporated into clinical diagnostics in nephrology. For example, mRNA abundance is increasingly being used as an adjunct to routine evaluation of transplant kidney biopsies and will be evaluated as a potential clinical trial endpoint in kidney transplantation [12]. Understanding the molecular underpinnings of LN could facilitate the development of personalized therapeutic strategies and identify novel therapeutic targets [13] and identify non-invasive promising biomarkers that could be employed in the management of patients with LN [14].

As a first step towards a molecular classification of LN, we evaluated the transcriptomic changes associated with four histologic phenotypes in a discovery cohort of 51 lupus patients at the University of Toronto (Toronto Cohort), and used an in silico approach to identify the biologic pathways correlated with histological and clinical phenotypes in patients with LN. A subset of transcripts correlating with histological phenotypes was evaluated in an independent cohort of 36 LN patients (Longitudinal Cohort) to determine how they changed over time and with disease activity.

## 2. Experimental Section

### 2.1. Materials and Methods

#### Experimental Design and Sample Collection

For the discovery Toronto Cohort, kidney biopsy material was obtained from patients undergoing a for-cause kidney biopsy from January 2007 to December 2012. With informed consent, a section of the core was stored in RNA-later (Life Technologies, Carlsbad, CA, USA) at 4 °C. Once determined that this tissue was not required for clinical diagnosis, the tissue was available for research (REB# 05-0759).

The Longitudinal Cohort is a unique independent cohort of patients that underwent serial kidney biopsies at The Hospital Fernandez (Buenos Aires, Argentina) between 2007 and 2018. This cohort is being used by researchers at Ohio State University to assess the evolution of LN over time. Data from this cohort have been previously described [15,16]. Briefly, LN patients underwent a for-cause kidney biopsy followed by a follow-up protocol kidney biopsy. All biopsy cores were formalin-fixed and paraffin-embedded (FFPE), and tissue remaining after clinical use was approved for research use by the Hospital Fernandez ethics board and The Ohio State University institutional review board (IRB# 2011H0364).

LN was classified by ISN/RPS criteria [3], and activity and chronicity were scored using the NIH system [8,17]. Lesions of interest (endocapillary hypercellularity, tubulointerstitial fibrosis, and cellular crescents) were graded on a semi-quantitative scale of 0–3 (0 = absent, 1 = mild, 2 = moderate, 3 = severe). A glomerulosclerosis score (0–4) was calculated by adding the score for segmental sclerosis (0 = absent, 1 = present) to that of global sclerosis (0 = absent, 1 = mild, 2 = moderate, 3 = severe). All biopsies were evaluated by a single renal pathologist for each cohort blinded to clinical information (CA-C for the Toronto Cohort and VA for the Longitudinal Cohort).

### 2.2. Sample Handling and RNA Isolation

RNA from the Toronto Cohort was extracted from micro-dissected glomerular and TI compartments, as previously described [18,19]. Following amplification (NuGEN, San Carlos, CA, USA) and reverse transcription (per Affymetrix GeneChip protocol), cDNA was hybridized to Affymetrix Human Transcriptome 2.0 arrays (Affymetrix, Santa Clara, CA, USA) and processed according to the manufacturer’s instructions.

RNA from the Longitudinal Cohort was extracted from laser-micro-dissected formalin-fixed paraffin-embedded (FFPE) kidney biopsies, as previously described [15]. Transcript abundance was analyzed using the NanoString nCounter^®^ GX human Immunology Panel (https://www.nanostring.com/products/gene-expression-panels/ncounter-immunology-panels, NanoString Technologies, Seattle, WA, USA). The panel consists of 579 immune response genes, 6 positive controls, 6 negative controls, and 15 housekeeping genes.

### 2.3. Microarray Processing and Statistical Analysis—Discovery Toronto Cohort

**Figure 1:**
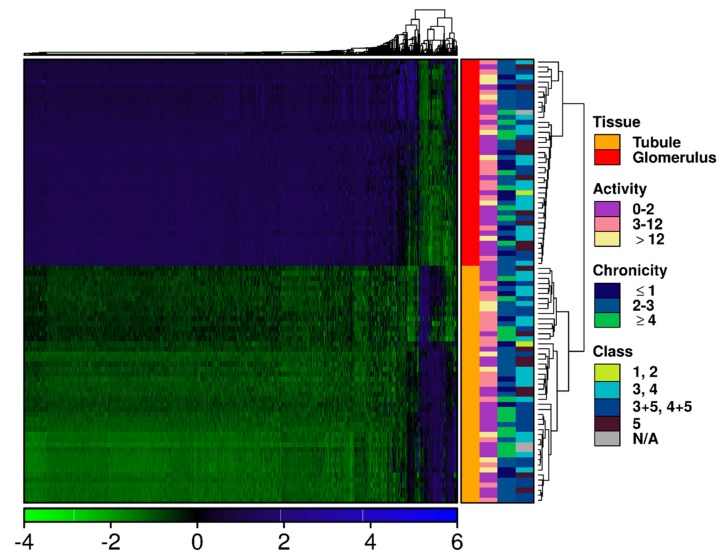
Glomeruli and tubulointerstitium display divergent transcriptional profiles. Transcripts with the most variable abundance levels across the dataset were isolated. Clustering of both transcripts and samples was performed using the DIANA hierarchical clustering algorithm, with Pearson’s correlation as a similarity metric. Each column represents scaled mRNA abundance level of a single transcript, while each row represents a sample. Covariates indicate tissue compartment (glomerular vs. tubulointerstitial), categories of the NIH (National Institute of Health) activity and chronicity indices and ISN/RPS (International Society of Nephrology/Renal Pathology Society) class.

Raw microarray data (CEL files) were loaded into the R statistical environment (v3.3.1) using the affy package (v1.48.0) of the BioConductor library [20]. Probes were mapped using the EntrezGene ID map hta20hsentrezgcdf (v20.0.0) (obtained from http://brainarray.mbni.med.umich.edu/) [21]. Raw data were processed using the RMA algorithm [22], handling each tissue compartment (glomerular vs. interstitial) separately to avoid masking compartment-specific mRNA abundance differences. Specifically, for each compartment, raw intensity values were background corrected, and sample distributions adjusted using quantile normalization. Raw and processed data are available from GEO (GSE127797). Data visualization was performed before and after normalization to assess array quality, with visualization performed using the BPG package (v5.9.8; PMID: 30665349 [23]), utilizing the lattice (v0.20–34) and latticeExtra (v0.6–28) packages for R. After normalization, glomerular and TI data were combined, and the variance among normalized intensity values for each gene determined across all samples. To visualize patterns in mRNA abundance, genes with a variance >2 were selected, and normalized intensity values were mean-centered and SD-scaled. A heatmap was generated for these genes only, such that both genes and samples were clustered using the DIANA hierarchical algorithm with Pearson’s correlation as a similarity metric using the cluster (v2.0.5) package (Figure 1). Unscaled normalized intensity values were used for all further analyses.

A dual approach was used to identify associations between mRNA abundance and disease metrics. For the first approach (hereby referred to as the binary approach), histologic lesions were considered as present/absent (presence/absence of endocapillary hypercellularity, tubulointerstitial fibrosis, cellular crescents, and glomerulosclerosis), and a Wilcoxon Rank Sum test was used to evaluate differential mRNA abundance for each gene between these groups, for each tissue type. This test evaluates the alternative hypothesis that the samples in each group come from the same population, such that randomly selected values from each group will have the same distribution. Here, for each gene, fold-change was calculated as the difference in median normalized RNA abundance (log2-space) between groups of interest. In the absence of statistically significant differential abundance at the preset threshold of false discovery rate (FDR)-adjusted *p*-value < 0.05, exploratory analyses using a more relaxed dual threshold of unadjusted *p* < 0.01 and |mRNA abundance difference| > 0.5 were used to identify sub-threshold transcripts with differentially abundant mRNA.

To complement this analysis, a second approach (hereby referred to as the semi-quantitative approach), Spearman’s rank correlation was used to measure the strength of association between transcript abundance and histological metrics of disease (including endocapillary hypercellularity, tubulointerstitial fibrosis, cellular crescents, and glomerulosclerosis). A threshold |Spearman’s ρ| > 0.5 was used to identify genes with mRNA abundance significantly correlated with each histological metric. Transcript abundance of genes identified in both statistical approaches was correlated to clinical parameters that represent disease activity (24 h proteinuria and serum creatinine) using Spearman’s rank correlation, with an FDR corrected *p*-value of 0.05 as a threshold for significance.

### 2.4. NanoString Statistical Analysis (Longitudinal Cohort)

Scatterplots and boxplots were used to visualize the data range and distribution. To adjust for variation in abundance that may arise from pipetting errors, instrument scan resolution, lot-to-lot variation in prep plates, and cartridges variation in purification, raw counts were technically normalized in the following manner; first, a correction factor was calculated according to the formula:

Correction factor for sample i = mean abundance of positive controls of reference sample/mean abundance of positive controls in sample i
(1)

The reference sample was chosen at random (with less than 1% variation when a different random reference sample was chosen). Then, for each sample, mRNA abundance was multiplied by the sample’s correction factor. Corrected raw NanoString counts were then log2 transformed. To reduce technical bias, genes with very low mRNA abundance (defined as NanoString counts below the mean plus two standard deviations of negative controls in more than 80% samples) were excluded. Quantile normalization was applied to NanoString counts of the remaining genes (522 for glomeruli and 502 for the tubulointerstitium) using the preprocessCore Bioconductor package [24]. To identify differentially-abundant mRNA from the first biopsy (Bx1) to the second biopsy (Bx2), linear mixed effect models were used, taking into account the correlation between repeated measures before and after treatment. For each biopsy, glomerular samples were analyzed separately from TI samples. To improve the stability of variance estimation, variance smoothing methods and moderated *t*-tests were employed [25]. Multiple hypothesis testing correction was done using the Bonferroni method (adjusted *p* < 0.01 considered significant). For any specific mRNA transcript to be considered differentially abundant, at least a |log2 (fold-change)| > 0.5 in abundance level and an adjusted *p*-value < 0.01 had to be achieved.

### 2.5. Clinical Outcome

Samples were stratified by the clinical renal response. A complete response (CR) was defined as an improvement in proteinuria to less than 0.5 g/day with normalization of serum creatinine. A non-response (NR) was defined as less than 50% reduction in proteinuria or proteinuria that remained more than 3 g/day with stable or worsening serum creatinine.

### 2.6. Pathway Analysis

Genes reaching statistical significance were uploaded to Interferome v2.0 [26] to identify interferon-regulated genes (IRGs). Genes identified in each individual analysis (dual cutoff of *p* < 0.01 and |median difference in abundance| > 0.5, or |Spearman’s ρ| > 0.5) were used to perform pathway analyses using the Enrichr platform [27] and utilizing the Reactome 2016 database [28]. Enrichr *p*-values were based on the Fisher exact test, *p*-values were then adjusted for multiple hypothesis testing using the Benjamini-Hochberg method. Then, using a lookup table of expected ranks of expression variances, a *z*-score was computed for deviation from this expected rank. The two scores were combined to produce a combined score (c = loge (p)·z). The top 25 pathways for each analysis can be found in the supplementary material.

## 3. Results

### 3.1. The Molecular Profiles of LN Kidney Biopsies

The Toronto Cohort of 51 patients was used to identify candidate molecular signatures from kidney tissue; cohort characteristics are provided in Table 1. The majority of patients were young (median age 33 years) and female (86%), with preserved renal function (median creatinine 68.5 µmol/L) and significant proteinuria (median 2.5 g/day). Each kidney biopsy was classified according to ISN/RPS criteria (Table 2); the majority demonstrated proliferative (35%), mixed proliferative and membranous (39%), and pure membranous (18%) lesions.

Following microdissection, RNA was available from both glomerular and TI compartments in 34 samples. In 13, RNA was available only from the TI compartment and, in seven, only from the glomerular compartment. The glomerular and TI compartments were distinguishable based upon the variable abundance of 1233 transcripts (variance > 2, Adjusted Rand Index (ARI) = 1) following divisive hierarchical clustering.

No statistically significant relationship was observed between transcript abundance and ISN/RPS class (ARI = −0.013) or NIH activity (ARI = −0.01) and chronicity (ARI = −0.012) indices based on these clusters (Figure 1).

Given the absence of a relationship between transcript abundance and the histopathologic class, we next related mRNA abundance with individual LN histologic features using two approaches. First, the difference in mRNA abundance according to the presence or absence of the lesion of interest was evaluated, using a binary analysis. Second, compartment-specific mRNA abundance was correlated with the severity of these lesions, as scored by a renal pathologist using a semi-quantitative scale (semi-quantitative approach; endocapillary hypercellularity (0–3), TI fibrosis (0–3), cellular crescents (0–3), and glomerulosclerosis (0–4)).

### 3.2. Correlates of Glomerular Inflammation

**Figure 2:**
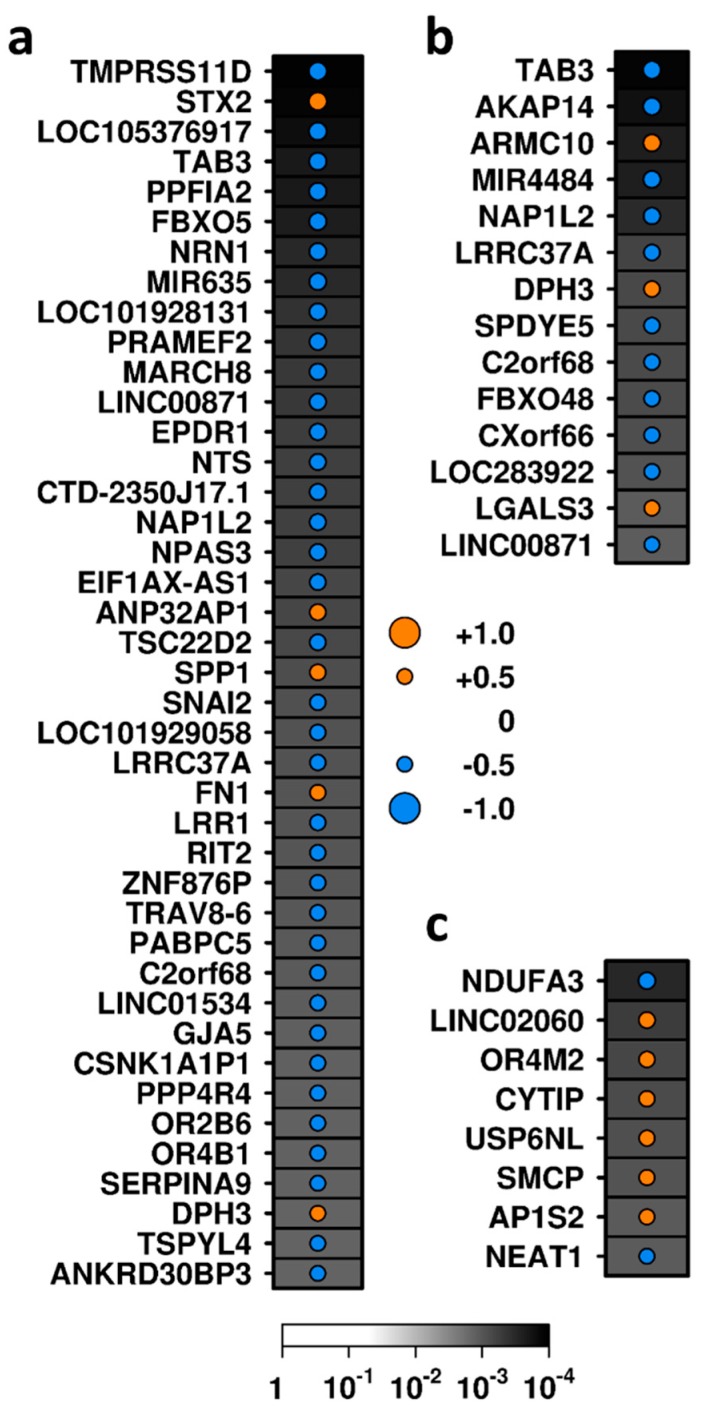
Exploratory analyses: transcripts demonstrating correlations with metrics of activity and glomerular scarring using the semi-quantitative approach. Transcripts correlated with (**a**) endocapillary hypercellularity, (**b**) crescent formation, (**c**) Glomerulosclerosis score. Dot size indicates the size of the correlation (Spearman’s ρ); color indicates the direction of correlation; background shading indicates the significance level (unadjusted *p*-value). Genes are ordered according to the absolute value of correlation statistic (ρ).

To identify molecular signatures of inflammatory and proliferative activity, we profiled two representative inflammatory lesions: endocapillary hypercellularity and cellular crescents. There were no statistically significant differentially abundant transcripts detected after correction for multiple hypothesis test (FDR-adjusted *p*-value < 0.05). We, therefore, performed exploratory analyses of subthreshold hits using a relaxed dual threshold of unadjusted *p* < 0.01 and |mRNA abundance difference| > 0.5 identified genes with differentially abundant mRNA. A total of 222 glomerular mRNA transcripts were differentially abundant between biopsies with and without endocapillary hypercellularity using this subthreshold *p*-value (Supplemental material), of which three showed median mRNA abundance differences >0.5 (*RAP1B*, *SPP1* showing increased abundance, and *SNORD42B* with lower abundance; Table S1). When endocapillary hypercellularity was semi-quantitatively graded, 330 mRNA transcripts exhibited sub-threshold correlations with the level of hypercellularity (unadjusted *p* < 0.01; Supplementary material), 36 of which were strongly negatively correlated (Spearman’s ρ < −0.5), while five showed strong positive correlations (Spearman’s ρ > 0.5; Table S1, Figure 2a). *SPP1* was associated with hypercellularity by both exploratory approaches, making it an interesting candidate for further study. The abundance of 189 glomerular transcripts exhibited subthreshold differences in mRNA abundance depending upon presence or absence of crescents (unadjusted *p* < 0.01; Supplemental material), and further applying a threshold of median mRNA abundance difference of 0.5 reduced this to nine transcripts. Of these, three (*ASF1A*, *HSPA5*, and *TRC-GCA4-1*) showed higher mRNA abundance in samples with crescents than those without, while the other six (*TRGJP2*, *C19orf73*, *RASA4B*, *SNORD54*, *SNORD4A*, and *LINC02297*) showed lower levels (Table S1) using exploratory analyses. Using the semi-quantitative approach, 159 transcripts exhibited subthreshold correlations with a crescent score (unadjusted *p* < 0.01; Supplemental material). Three of these were strongly positively correlated (Spearman’s ρ > 0.5) and 11 were strongly negatively correlated (Spearman’s ρ < −0.5) with a crescent score (Table S1, Figure 2b). Transcripts representing five genes (*C2orf68*, *LINC00871*, *LRRC37A*, *NAP1L2*, and *TAB3*) were negatively correlated with both endocapillary hypercellularity and crescents, while one (*DPH3*) was positively correlated with both (|Spearman’s ρ| > 0.5).

Pathway analysis of subthreshold mRNA transcripts associated with endocapillary hypercellularity or crescents using these approaches was performed (Table S2). This analysis identified pathways implicated in cellular proliferation and endothelial injuries, such as p53 mitogen-associated protein kinases (MAPK) signaling, nuclear factor kappa B (NFκB) activation, integrin signaling, innate immune system signaling, and extracellular matrix synthesis.

### 3.3. Correlates of Tubulointerstitial Fibrosis and Glomerulosclerosis

**Figure 3:**
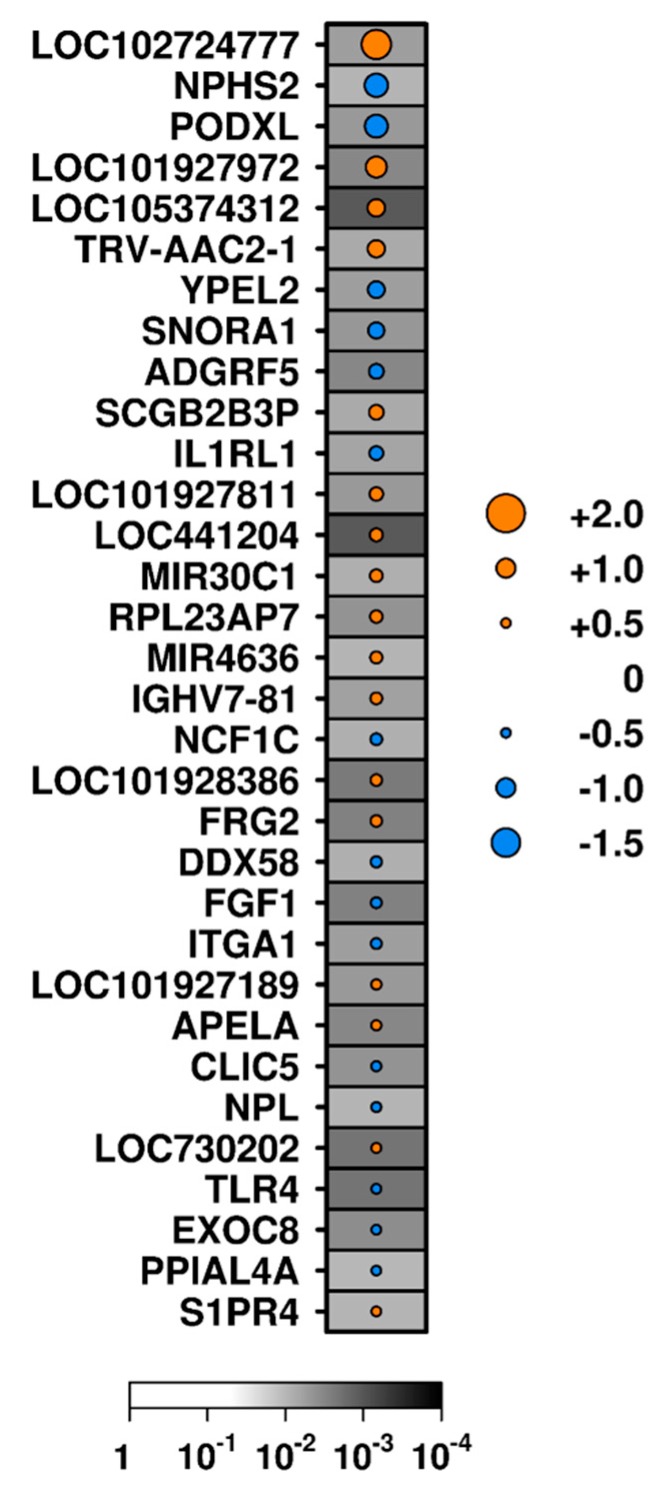
Exploratory analysis: transcripts with differential abundance in patients with or without interstitial fibrosis. Dot size indicates the median difference in RNA abundance; color indicates the direction of difference (orange indicates an increase in samples with fibrosis relative to those without, while blue indicates a decrease); background shading indicates significance level (unadjusted *p*-value). Genes are ordered by the magnitude of difference.

Tubulointerstitial fibrosis is an irreversible marker of disease chronicity and is generally a marker of poor long-term prognosis. Exploratory analysis of subthreshold hits identified 101 transcripts with different mRNA abundance between tubulointerstitium samples with and without interstitial fibrosis (Supplemental material) using a binary approach, including 15 and 17 that showed increased or decreased RNA abundance, respectively (unadjusted *p* < 0.01 and |median RNA abundance difference| > 0.5; Table S1, Figure 3). Abundance of 59 genes with RNA correlated with fibrosis score (unadjusted *p* < 0.01; Supplemental material); however, only two of these (*FRG2* and *LOC441204*) had a Spearman’s ρ > 0.5, while one (*CTTNBP2*) was negatively correlated with fibrosis score (Spearman’s ρ < −0.5; Table S1). *FRG2* and *LOC441204* were identified by both the binary and semi-quantitative approaches and, therefore, warrant further study.

Glomerulosclerosis is an irreversible lesion thought to occur as glomerular inflammation heals. The exploratory analysis identified 62 transcripts with subthreshold differential RNA abundance between biopsies with or without glomerular sclerosis (unadjusted *p* < 0.01; Supplemental material). One gene (*OR4M2*) showed higher RNA abundance, while two genes (*SCARNA9* and *TP53TG1*) had lower RNA abundance (difference > 0.5) in samples with sclerosis than those without (Table S1). The abundance of 74 transcripts correlated with glomerulosclerosis score (unadjusted *p* < 0.01 Supplemental material), eight of which had stronger correlation (|Spearman’s ρ| > 0.5) (Table S1, Figure 2c), and *OR4M2* was associated with sclerosis by both analytic approaches.

Pathway analysis of transcripts associated with glomerulosclerosis and TI fibrosis revealed the involvement of fibroblast growth factor signaling, nuclear factor kappa B (NFκB) activation, vesicular transport, and immunomodulation by viral particles (Table S3).

To determine whether there was an interferon signature associated with disease activity, we used Interferome^®^ [26] to identify interferon-regulated-genes (IRGs). In the Toronto Cohort, 44 out of the 102 genes with mRNA abundance that were associated with the four histologic lesions by exploratory analyses (endocapillary hypercellularity, crescent formation, glomerulosclerosis, and tubulointerstitial fibrosis) were IRGs.

### 3.4. Correlates of Clinical Variables

The 102 transcripts where mRNA abundance was associated with the four histologic lesions studied in exploratory analyses were also correlated with two additional clinical parameters: 24-h urine protein and serum creatinine. Tubulointerstitial mRNA abundance of fibronectin (*FN1*; ρ = 0.579, FDR-adjusted *p*-value = 0.004) and *LINC00871* (ρ = −0.505, FDR-adjusted *p*-value = 0.028) were significantly correlated with serum creatinine levels (Supplemental material). None correlated with 24-h proteinuria using FDR-adjusted *p*-value threshold of <0.05.

### 3.5. Evolution of Transcript Abundance after Treatment

The Longitudinal Cohort afforded the opportunity to characterize how kidney transcripts change with treatment in patients who respond or do not respond to therapy. It included 36 patients who had a diagnostic kidney biopsy because LN was suspected (Bx1) and that was repeated after induction therapy (Bx2). These patients are described in Table S4. All were receiving hydroxychloroquine at Bx1, but no other immunosuppression. After a diagnosis of LN, patients were treated with a tapering course of prednisone starting at 1 mg/kg/day and either mycophenolate mofetil or intravenous cyclophosphamide. After induction therapy, at the time of Bx2, patients were assessed clinically for renal remission and stratified by response into complete responders and non-responders.

The treatment-associated changes in abundance of transcripts identified in our exploratory analyses in the Toronto Cohort (unadjusted *p* < 0.01 and |difference in RNA abundance| > 0.5 or |Spearman’s ρ| > 0.5) (*n* = 102 transcripts, Table S1) were evaluated in serial biopsies in the Longitudinal Cohort. Given that the Longitudinal Cohort analysis was designed for an independent study [15,16], not all transcripts identified in the discovery Toronto Cohort were represented in the NanoString derived dataset. A subset of five was included in the NanoString Immunology Panel (*FN1*, *SPP1*, *LGALS3*, *IL1RL1*, *TLR4*, which encode the proteins fibronectin, galectin-3, interleukin-1 receptor like-1, and toll-like receptor 4, Table 3). Comparing changes in mRNA abundance between Bx1 to Bx2, glomerular *FN1* RNA abundance significantly increased in patients, who did not respond to treatment (log2 (fold-change) = 1.47, *p* = 0.002, Bonferroni critical *p* < 0.01), and significantly decreased in patients, who achieved CR (log2 (fold-change) = −0.96, *p* = 0.0011, Bonferroni critical *p* < 0.01). In addition, glomerular abundance of *SPP1* significantly decreased in patients achieving CR (log2 (fold-change) = –1.77, *p* = 0.0016, Bonferroni critical *p* < 0.01), but did not change in patients with NR. The glomerular abundance of LGALS3 RNA was decreased in patients achieving CR (log2 (fold-change) = −0.47, *p* = 0.0296, Bonferroni critical *p* < 0.01, subthreshold analysis) and did not change in patients with NR.

In TI tissue of patients in the Toronto Cohort, *IL1RL1* and *TLR4* mRNA transcripts were less abundant in patients with interstitial fibrosis than those without (Figure 3). RNA abundance of these transcripts did not change in patients with CR or NR between B×1 to Bx2 (Table 3) in the Longitudinal Cohort.

## 4. Discussion

The histological phenotypes of LN have long been used to guide disease management, but the molecular pathophysiology of specific renal lesions is still poorly understood. Furthermore, histologic classification schemes do not consistently predict the outcome or treatment response. This study identified several molecular processes that are associated with specific histologic features and may contribute to their pathogenesis. Furthermore, a subset of these markers demonstrated changes in mRNA abundance following therapeutic intervention.

Our first major finding was that the correlations between compartment-specific tissue mRNA abundance and ISN/RPS histologic classification were too weak to be detected, at least given our cohort size and makeup. The ISN/RPS classification scheme was not derived based upon pathogenic molecular pathways; therefore, this highlighted a potential role for molecular data to add independent diagnostic and prognostic information beyond conventional class. However, when segregated by individual histologic lesions, several subthreshold (using an unadjusted *p* < 0.01) candidate histologic-molecular correlates emerged by exploratory analysis, many reflecting interferon regulated genes (IRGs). This IRG bias was also observed and previously reported in an independent LN population (the Longitudinal Cohort) [16], where type I interferon gene abundance was decreased after treatment in patients achieving a CR and increased in patients without a response to treatment. These findings were in line with previous studies demonstrating increased IRG expression in active SLE [29,30], and were also supported by the observed reduced disease activity in SLE patients after inhibiting type I interferon (IFN) activity by blocking type I IFN-α/β/ω receptor (IFNAR) with anifrolumab [31], which is being evaluated in LN (ClinicalTrials.gov Identifier: NCT02547922).

Our molecular analysis of individual kidney compartments was consistent with years of work on injury pathways in LN. In particular, the transcript for osteopontin (*SPP1*) demonstrated both a significant correlation with cellular proliferation in the Toronto Cohort and a significant decrease in its abundance in patients achieving CR in the Longitudinal Cohort. Osteopontin is a secreted and intracellular phosphoprotein that has been linked to autoimmunity and SLE. It has multiple roles and is involved in INF-α signaling, Th17 cell regulation, and inhibition of apoptotic cell clearance by macrophages, thus potentially activating autoreactive B cells [32,33]. It is also involved in the actions of plasmacytoid dendritic cells (pDC) and alternatively-activated macrophages, the latter of which have been directly linked to cellular proliferation and crescent formation in murine LN [34]. More recently, higher circulating osteopontin levels were observed in SLE patients compared to controls and were identified as a marker for increased cumulative disease activity and organ damage in SLE patients [32]. In addition, glomerular osteopontin abundance was increased in 22 cases of crescentic glomerulonephritis (including one with class IV LN) when compared to disease controls, which did not demonstrate any glomerular osteopontin abundance [35]. The majority of osteopontin-secreting cells were identified as macrophages/monocytes. More interestingly, the majority of normal-appearing glomeruli did not contain any cells expressing osteopontin; thus, osteopontin warrants further evaluation as a potential marker of aggressive and proliferative disease.

Another IRG identified was *LGALS3* (which encodes galectin-3). It positively correlated with the crescent formation in the Toronto Cohort and showed reduced abundance in patients achieving CR in the Longitudinal Cohort. Previous studies demonstrated an increase in *LGALS3* abundance in LN glomeruli, and a positive correlation with multiple serologic and histologic features, such as anti-dsDNA titers, cellularity, cellular crescents, and fibrinoid necrosis, and a negative correlation with complement 3 and 4 levels [36]. Moreover, galectin-3 binding protein (G3BP) has a role in an immune-complex deposition in LN [37], and SLE patients have increased levels of G3BP reflecting type I interferon activity [38]. Thus, *LGALS3* also deserves evaluation as a marker of aggressive LN.

Some of the transcripts that correlated with histological markers of activity in the Toronto Cohort also manifested significant changes in abundance profile according to clinical disease activity in the Longitudinal Cohort. In addition to *SPP*1 and *LGALS3* described above, glomerular abundance of *FN1* (which encodes fibronectin) correlated with cellular proliferation and serum creatinine level in the Toronto Cohort. Its abundance significantly decreased following treatment in patients with CR and increased in patients with NR in the Longitudinal Cohort [16]. Taken together, these findings support glomerular fibronectin, galectin-3, and osteopontin abundance as potential biomarkers of LN activity that can represent histological and clinical changes.

Pathway analyses of transcripts associated with lesions of endocapillary hypercellularity and crescent formation demonstrated similar themes, including activation of MAPK and NFκB signaling, both of which are implicated in the regulation of renal inflammation [39,40]. The contribution of the MAPK system in SLE is mediated by the p38 and extracellular signal-regulated kinase (ERK) subfamilies. The contribution of the ERK pathway to SLE was suggested to be mediated via its effects on T cell DNA methylation [41]. In addition, blocking the activation of p38 MAPK and NFκB by CD8+-induced regulatory T-cells can attenuate endothelial cell injury [42], suggesting an important contribution of T cells in those histological lesions of activity. Alternatively, the pathway analysis of transcripts involved in interstitial fibrosis demonstrated the activation of several fibroblast growth factor (FGF) receptors. FGF-receptors (FGFR) 1–4 are high-affinity receptors of FGF-2, which induce proliferation of human fibroblasts stimulated by transforming growth factor-beta (TGFβ) [43]. In addition, the tyrosine kinase receptor inhibitor nintedanib, which blocks FGFR, platelet-derived growth factor receptor (PDGFR), vascular endothelial growth factor receptor (VEGFR), and Src family kinases, has been FDA approved for the treatment of pulmonary fibrosis and was found to attenuate renal fibrosis and inhibit activation of renal interstitial fibroblasts in a mouse model of renal fibrosis [44]. Finally, pathway analysis of transcripts involved in glomerular sclerosis demonstrated the involvement of processes related to vesicular transport and trafficking and viral immunomodulation. This finding is not surprising as abnormalities in vesicular transport (and clathrin-mediated endocytosis specifically) have been implicated in podocytopathies that manifest histologically with the focal and segmental glomerular sclerosis lesion [45]. A relationship between retroviruses and proliferative LN was first suggested in the 1970s when the retroviral p30 gag protein was detected in glomerular immune deposits [46]. Moreover, endogenous retrovirus elements (ERVs) and other retrotransposable elements make up a large part of the human genome [47].

Despite the abundance of intriguing findings presented here, we acknowledge this study has several limitations. Compartmental transcriptomics has limited insight into the role of specific cell-types and their contribution to disease. In addition, the biopsies were only evaluated by one pathologist that can affect classification and scoring. The majority of the patients in the Toronto Cohort were receiving immunosuppressive therapy at the time of biopsy, and thus the identified molecular processes didn’t represent treatment-naïve LN. In addition, neither cohort had a significant number of patients with mild mesangial disease (ISN/RPS class I/II); thus, the different molecular processes that characterize mild versus severe disease could not be identified, and there were considerable differences in patient characteristics, tissue preservatives, and analysis platforms among our cohorts. In particular, the NanoString panel used is enriched for genes involved in autoimmunity. Moreover, the cross-sectional nature of the Toronto Cohort does not necessarily reflect the molecular processes that were active while the histological lesions were evolving. Nonetheless, the cross-sectional findings from the Toronto Cohort overlapped modestly with the findings from the Longitudinal Cohort, providing some external data validation and linking molecular processes to histological and clinical findings. In addition, the limited correlations between molecular and histological findings suggest that relying solely on a histological classification of LN may not be the best approach as it misses valuable information that is gained from molecular analysis. The findings from the Longitudinal Cohort also support a similar premise and suggest that molecular data, such as the interferon gene signature, might be more representative of disease activity as it links histological and clinical markers of activity.

To conclude, this study is a first step towards characterizing LN on a molecular basis that will pave the way towards a more comprehensive disease classification, the identification of new biomarkers, and therapeutic targets.

## Figures and Tables

**Table 1:** Clinical profiles of patients from the Toronto Cohort. Values displayed as mean ± SD or median (IQR, interquartile range) as appropriate.

Age (Years)	33 (24–45)
Sex	*n* (%)
Female	44 (86)
Male	7 (14)
Creatinine (µmol/L) *	68.5 (59.5–97.0)
Proteinuria (g/d) **	2.5 (1.5–5)
Mean Arterial Pressure (mm Hg) *	98.8 ± 14.5
Therapeutic Intervention	*n* (%)
Hydroxychloroquine	30 (56)
RAAS Blocker	22 (41)
Any Immunosuppression	41 (76)
Prednisone alone	16 (30)
Prednisone plus	
MMF	11 (20)
AZT	11 (20)
CNI ^†^	3 (6)
MTX ^†^	1 (2)

Abbreviations: RAAS: Renal angiotensin aldosterone system. MMF: Mycophenolate mofetil/sodium, AZT: Azathioprine. CNI: Calcineurin inhibitor. MTX: Methotrexate. * Value closest to the time of biopsy; ** Value closest to time of biopsy; ^†^ One patient was receiving a combination of prednisone, CNI, and MTX. Another patient was receiving MTX monotherapy.

**Table 2:** Histological features on kidney biopsy of patients from the Toronto Cohort. Values displayed as mean ± SD or median (IQR) as appropriate. International Society of Nephrology (ISN), Renal Pathology Society (RPS).

Histological Feature	*n* (%)
ISN/RPS Class	
I + II	2 (4)
III–IV	19 (35)
III–IV + V	21 (39)
V	10 (18)
VI	2 (4)
NIH Indices	
Activity Index (max 24)	3.5 (1–9)
Chronicity Index (max 12)	3 (2–4)
Histological Lesions	
Cellular Proliferation	33 (61)
1	12
2	15
3	6
Crescents	24 (44)
1	10
2	9
3	5
Interstitial Fibrosis	45 (83)
1	35
2	6
3	4
Any Sclerosis	39 (72)
Segmental	26 (48)
Global	32 (59)
1	20
2	6
3	6

**Table 3:** Differentially abundant transcripts observed in both cohorts.

Compartment	Transcript	Toronto Cohort	Longitudinal Cohort	
			Complete Responders	Non-Responders
Glomerular	*FN1*	Positive correlation with the degree of endocapillary hypercellularity	Decreased with response	Increased
	*LGALS3*	Positive correlation with degree of crescent formation	Trend towards decrease with response	No change
	*SPP1*	Positive correlation with the degree of endocapillary hypercellularity	Decreased with response	No change
Tubulointerstitial	*IL1RL1*	Decreased level in patients with interstitial fibrosis	No Change	No change
	*TLR4*	Decreased level in patients with interstitial fibrosis	No Change	No Change

The abundance of glomerular *FN1*, *LGALS3*, and *SPP1* RNA was concordant in both cohorts and reflective of activity. All five genes were interferon-regulated.

## Supplementary Materials

The following are available online at https://www.mdpi.com/2077-0383/8/10/1524/s1, Table S1: Exploratory analyses of differential abundance according to dual analytic approaches, Table S2: Exploratory pathway analysis of describing histological lesions of activity. Endocapillary hypercellularity in (a), crescent formation in (b), Table S3: Exploratory pathway analysis of transcripts describing histological lesions of chronic damage. Interstitial fibrosis in (a), global and segmental sclerosis (b), Table S4: Clinical profiles of patients from the Longitudinal Cohort. Values displayed as mean ± SD or median (IQR) as appropriate.
