# Supplementary material for: Rethinking Lupus Nephritis Classification on a Molecular Level
Source: J Clin Med. 2019 Sep 23;8(10):1524. doi: 10.3390/jcm8101524 (PMC6832959; doi:10.3390/jcm8101524)
Supplement: Supplementary file 1 [file jcm-08-01524-s001.pdf]

Table S1. Exploratory analyses of differential abundance according to dual analytic approaches*.

Cellular proliferation (glomerular compartment)			Interstitial fibrosis (tubulointerstitial compartment)		
Binary	Semi-quantitative		Binary		Semi-quantitative
SPP1	TMPRSS11D	SNAI2	LOC102724777	FGF1	LOC441204
RAP1B	STX2	LOC101929058	NPHS2	ITGA1	FRG2
SNORD42B	LOC105376917	LRRC37A	PODXL	LOC101927189	CTTNBP2
	TAB3	FN1	LOC101927972	APELA	
	PPFIA2	LRR1	LOC105374312	CLIC5	
	FBXO5	RIT2	TRV-AAC2-1	NPL	
	NRN1	ZNF876P	YPEL2	LOC730202	
	MIR635	TRAV8-6	SNORA1	TLR4	
	LOC101928131	PABPC5	ADGRF5	EXOC8	
	PRAMEF2	C2orf68	SCGB2B3P	PPIAL4A	
	MARCH8	LINC01534	IL1RL1	S1PR4	
	LINC00871	GJA5	LOC101927811		
	EPDR1	CSNK1A1P1	LOC441204		
	NTS	PPP4R4	MIR30C1		
	CTD-2350J17.1	OR2B6	RPL23AP7		
	NAP1L2	OR4B1	MIR4636		
	NPAS3	SERPINA9	IGHV7-81		
	EIF1AX-AS1	DPH3	NCF1C		
	ANP32AP1	TSPYL4	LOC101928386		
	TSC22D2	ANKRD30BP3	FRG2		
	SPP1		DDX58		

Crescent formation (glomerular compartment)			Sclerosis score (glomerular compartment)	
Binary	Semi-quantitative		Binary	Semi-quantitative
ASF1A	TAB3	FBXO48	SCARNA9	NDUFA3
HSPA5	AKAP14	CXorf66	TP53TG1	LINC02060
TRGJP2	ARMC10	LOC283922	OR4M2	OR4M2
C19orf73	MIR4484	LGALS3		USP6NL
RASA4B	NAP1L2	LINC00871		SMCP

SNORD54	LRRC37A		AP1S2	
SNORD4A	DPH3		NEAT1	
TRC-GCA4-1	SPDYE5			
LINC02297	C2orf68			

* The lists represent all transcripts (n=102) considered in an exploratory analysis according to sub-threshold criteria of unadjusted p<0.01 and ∣median RNA abundance difference ∣>0.5 or ∣Spearman's ϱ∣ >0.5) in each of the analyses performed on samples from the Toronto Cohort. The transcripts in red are interferon regulated genes (46 out of 102).

Table S2. Exploratory pathway analysis of describing histological lesions of activity. Endocapillary hypercellularity in (a), crescent formation in (b).

(a) Endocapillary hypercellularity

	Pathway name	P-value	Adjusted p-value	Z-score	Combined score
1	GRB2:SOS provides linkage to MAPK signaling for Integrins_Homo sapiens_R-HSA-354194	0.0004658	0.03447	-2.3	17.62
2	p130Cas linkage to MAPK signaling for integrins_Homo sapiens_R-HSA-372708	0.0004658	0.03447	-2.15	16.46
3	Integrin alphaIIb beta3 signaling_Homo sapiens_R-HSA-354192	0.001532	0.07556	-2.13	13.83
4	MAP2K and MAPK activation_Homo sapiens_R-HSA-5674135	0.003022	0.08945	-2.3	13.33
5	Platelet Aggregation (Plug Formation)_Homo sapiens_R-HSA-76009	0.002867	0.08945	-1.95	11.43
6	Signalling to p38 via RIT and RIN_Homo sapiens_R-HSA-187706	0.01463	0.2297	-2.43	10.25
7	Signalling to ERKs_Homo sapiens_R-HSA-187687	0.01701	0.2297	-2.44	9.96
8	Integrin cell surface interactions_Homo sapiens_R-HSA-216083	0.009137	0.2254	-1.95	9.18
9	Signaling by PDGF_Homo sapiens_R-HSA-186797	0.0432	0.2492	-2.42	7.6
10	NGF signalling via TRKA from the plasma membrane_Homo sapiens_R-HSA-187037	0.04619	0.2492	-2.37	7.3
11	Fc epsilon receptor (FCERI) signaling_Homo sapiens_R-HSA-2454202	0.05279	0.2492	-2.33	6.85
12	Signaling by Interleukins_Homo sapiens_R-HSA-449147	0.05182	0.2492	-2.26	6.68
13	Synthesis of diphthamide-EEF2_Homo sapiens_R-HSA-5358493	0.01707	0.2297	-1.54	6.29
14	Fibronectin matrix formation_Homo sapiens_R-HSA-1566977	0.01283	0.2297	-1.43	6.24
15	Gastrin-CREB signaling pathway via PKC and MAPK_Homo sapiens_R-HSA-881907	0.06548	0.2492	-2.28	6.21

(b) Crescent formation					
	Pathway name	P-value	Adjusted p-value	Z-score	Combined score
1	Synthesis of diphthamide-EEF2_Homo sapiens_R-HSA-5358493	0.009165	0.1221	-2.41	11.33
2	ATF6-alpha activates chaperone genes_Homo sapiens_R-HSA-381183	0.01144	0.1221	-2.09	9.36
3	IRAK2 mediated activation of TAK1 complex upon TLR7/8 or 9 stimulation_Homo sapiens_R-HSA-975163	0.01144	0.1221	-1.76	7.89
4	TRAF6 mediated induction of TAK1 complex_Homo sapiens_R-HSA-937072	0.01825	0.1221	-1.94	7.76
5	JNK (c-Jun kinases) phosphorylation and activation mediated by activated human TAK1_Homo sapiens_R-HSA-450321	0.01825	0.1221	-1.93	7.71
6	IRAK2 mediated activation of TAK1 complex_Homo sapiens_R-HSA-937042	0.01144	0.1221	-1.72	7.7
7	ATF6-alpha activates chaperones_Homo sapiens_R-HSA-381033	0.01372	0.1221	-1.8	7.7
8	Formation of Senescence-Associated Heterochromatin Foci (SAHF)_Homo sapiens_R-HSA-2559584	0.01938	0.1221	-1.93	7.59
9	TAK1 activates NFkB by phosphorylation and activation of IKKs complex_Homo sapiens_R-HSA-445989	0.02949	0.1428	-2.06	7.24
10	Advanced glycosylation endproduct receptor signaling_Homo sapiens_R-HSA-879415	0.01485	0.1221	-1.7	7.16
11	activated TAK1 mediates p38 MAPK activation_Homo sapiens_R-HSA-450302	0.01938	0.1221	-1.77	7
12	NOD1/2 Signaling Pathway_Homo sapiens_R-HSA-168638	0.03507	0.1473	-2.07	6.94
13	PERK regulates gene expression_Homo sapiens_R-HSA-381042	0.03173	0.1428	-1.96	6.78
14	Antigen Presentation: Folding, assembly and peptide loading of class I MHC_Homo sapiens_R-HSA-983170	0.02837	0.1428	-1.77	6.32
15	TNFR1-induced NFkappaB signaling pathway_Homo sapiens_R-HSA-5357956	0.02949	0.1428	-1.76	6.21

Pathway analysis of transcripts that achieved statistical significance based on a threshold of $p<0.01$ and ∣median RNA abundance difference∣>0.5 ∣Spearman's ϱ∣ >0.5. Analysis was done using Enrichr and Reactome 2016 database. The top 15 pathways involved in endocapillary hypercellularity (a), and crescent formation (b) are displayed. Z-score indicates the Enrichr Z score. The combined score is calculated from the p-value obtained from Fisher's exact test and the Enrichr z-score (combined score =log(p)*z). Pathways are ordered based on the combined score in descending order.

.

Table S3. Exploratory pathway analysis of transcripts describing histological lesions of chronic damage. Interstitial fibrosis in (a), global and segmental sclerosis (b).

**(a) Interstitial fibrosis**					
	Pathway name	P-value	Adjusted p-value	Z-score	Combined score
1	FCERI mediated MAPK activation_Homo sapiens_R-HSA-2871796	0.02125	0.1656	-2.48	9.55
2	Axon guidance_Homo sapiens_R-HSA-422475	0.02075	0.1656	-2.3	8.93
3	Innate Immune System_Homo sapiens_R-HSA-168249	0.02382	0.1656	-2.3	8.61
4	VEGFA-VEGFR2 Pathway_Homo sapiens_R-HSA-4420097	0.02764	0.1656	-2.34	8.38
5	Signaling by VEGF_Homo sapiens_R-HSA-194138	0.02945	0.1656	-2.26	7.98
6	Downstream signal transduction_Homo sapiens_R-HSA-186763	0.0325	0.1656	-2.32	7.94
7	CHL1 interactions_Homo sapiens_R-HSA-447041	0.0183	0.1656	-1.92	7.69
8	Signaling by PDGF_Homo sapiens_R-HSA-186797	0.03831	0.1656	-2.23	7.27
9	MAPK1 (ERK2) activation_Homo sapiens_R-HSA-112411	0.0183	0.1656	-1.71	6.84
10	Cytokine Signaling in Immune system_Homo sapiens_R-HSA-1280215	0.03748	0.1656	-2.07	6.79
11	MAPK3 (ERK1) activation_Homo sapiens_R-HSA-110056	0.02032	0.1656	-1.72	6.72
12	FGFR1b ligand binding and activation_Homo sapiens_R-HSA-190370	0.01224	0.1656	-1.52	6.71
13	Interleukin-6 signaling_Homo sapiens_R-HSA-1059683	0.02233	0.1656	-1.66	6.33
14	Fc epsilon receptor (FCERI) signaling_Homo sapiens_R-HSA-2454202	0.04692	0.1656	-2.06	6.3
15	Signaling by Interleukins_Homo sapiens_R-HSA-449147	0.04604	0.1656	-2.03	6.26
**(b) Global and segmental sclerosis**					
	Pathway name	P-value	Adjusted p-value	Z-score	Combined score
1	Nef mediated downregulation of MHC class I complex cell surface expression_Homo sapiens_R-HSA-164940	0.004492	0.07213	-2.12	11.44
2	Nef-mediates down modulation of cell surface receptors by recruiting them to clathrin adapters_Homo sapiens_R-HSA-164938	0.009412	0.07213	-2.18	10.15
3	The role of Nef in HIV-1 replication and disease pathogenesis_Homo sapiens_R-HSA-164952	0.01253	0.07213	-2.31	10.13
4	Membrane Trafficking_Homo sapiens_R-HSA-199991	0.01436	0.07213	-2.12	9.01
5	Lysosome Vesicle Biogenesis_Homo sapiens_R-HSA-432720	0.01564	0.07213	-2.06	8.54
6	Vesicle-mediated transport_Homo sapiens_R-HSA-5653656	0.01939	0.07213	-2.12	8.38
7	Complex I biogenesis_Homo sapiens_R-HSA-6799198	0.02184	0.07213	-2	7.64
8	Golgi Associated Vesicle Biogenesis_Homo sapiens_R-HSA-432722	0.02404	0.07213	-1.9	7.07
9	Retrograde transport at the Trans-Golgi-Network_Homo sapiens_R-HSA-6811440	0.02184	0.07213	-1.78	6.82
10	Clathrin derived vesicle budding_Homo sapiens_R-HSA-421837	0.03107	0.07626	-1.78	6.19
11	Host Interactions of HIV factors_Homo sapiens_R-HSA-162909	0.05659	0.1019	-2.15	6.19
12	trans-Golgi Network Vesicle Budding_Homo sapiens_R-HSA-199992	0.03107	0.07626	-1.77	6.16
13	Respiratory electron transport_Homo sapiens_R-HSA-611105	0.03892	0.08757	-1.88	6.1
14	Respiratory electron transport, ATP synthesis by chemiosmotic coupling, and heat production by uncoupling proteins._Homo sapiens_R-HSA-163200	0.048	0.09258	-1.89	5.73
15	MHC class II antigen presentation_Homo sapiens_R-HSA-2132295	0.04542	0.09258	-1.84	5.7

Pathway analysis of transcripts that achieved statistical significance based on a threshold of $p<0.01$ and ∣median RNA abundance difference ∣>0.5 ∣Spearman's ϱ∣ >0.5. Analysis was done using Enrichr and Reactome 2016 database. The top 15 pathways involved in interstitial fibrosis (a), and global and segmental sclerosis (b) are displayed. Z-score indicates the Enrichr Z score. The combined score is calculated from the p-value obtained from Fisher's exact test and the Enrichr z-score (combined score =log(p)*z). Pathways are ordered based on the combined score in descending order.

Table S4. Clinical profiles of patients from the Longitudinal Cohort. Values displayed as mean ±SD or median (IQR) as appropriate.

	CR (n=27)	NR (n=9)	P value
Age (years)	30.9 ± 9	31.4 ± 10	NS
Female (%)	24 (89)	4 (55)	*0.02*
Class IV (%)	17 (63)	5 (56)	NS
MMF Induction (%)	14 (52)	3 (33)	NS
Serum creatinine (μmol/L)			
at Bx_1	61.9 ± 17.7	114.9 ± 88.4	*0.004*
at Bx_2	61.9 ±8.8	88.4 ± 39.8	*0.003*
Proteinuria (g/d)			
at Bx_1	3.0 ± 1.6	3.1 ± 3.0	NS
at Bx_2	0.2 ± 0.1	2.2 ± 1.7	*<0.0001*
Median time to Follow up (months)	8 (5-15)	7 (6-13)	NS
NIH indices:			
Activity Index (max 24)			
Bx_1	8 (2-15)	7 (4-12)	NS
Bx_2	3.5 (0-6)	3 (0-9)	NS
Chronicity Index (max 12)			
Bx_1	3 (0-6)	3 (0-6)	NS
Bx_2	4 (2-7)	4 (1-8)	NS
